# NKG2D promotes CD8 T cell-mediated cytotoxicity and is associated with treatment failure in human cutaneous leishmaniasis

**DOI:** 10.1371/journal.pntd.0011552

**Published:** 2023-08-21

**Authors:** Laís A. Sacramento, Camila Farias Amorim, Taís M. Campos, Maíra Saldanha, Sérgio Arruda, Lucas P. Carvalho, Daniel P. Beiting, Edgar M. Carvalho, Fernanda O. Novais, Phillip Scott

**Affiliations:** 1 Department of Pathobiology, School of Veterinary Medicine, University of Pennsylvania, Philadelphia, Pennsylvania, United States of America; 2 Serviço de Imunologia, Complexo Hospitalar Prof. Edgard Santos, Universidade Federal da Bahia, Salvador, Brazil; 3 Centro de Pesquisas Gonçalo Moniz, Fundação Oswaldo Cruz, Salvador, Brazil; 4 Instituto Nacional de Ciências e Tecnologia-Doenças Tropicais, Salvador, Brazil; 5 Department of Microbial Infection and Immunity, College of Medicine, The Ohio State University, Columbus,Ohio, United States of America; Pasteur Institute of Iran, ISLAMIC REPUBLIC OF IRAN

## Abstract

Cutaneous leishmaniasis exhibits a spectrum of clinical presentations dependent upon the parasites’ persistence and host immunopathologic responses. Although cytolytic CD8 T cells cannot control the parasites, they significantly contribute to pathologic responses. In a murine model of cutaneous leishmaniasis, we previously found that NKG2D plays a role in the ability of cytolytic CD8 T cells to promote disease in leishmanial lesions. Here, we investigated whether NKG2D plays a role in human disease. We found that NKG2D and its ligands were expressed within lesions from *L*. *braziliensis*-infected patients and that IL-15 and IL-1β were factors driving NKG2D and NKG2D ligand expression, respectively. Blocking NKG2D reduced degranulation by CD8 T cells in a subset of patients. Additionally, our transcriptional analysis of patients’ lesions found that patients who failed the first round of treatment exhibited higher expression of *KLRK1*, the gene coding for NKG2D, than those who responded to treatment. These findings suggest that NKG2D may be a promising therapeutic target for ameliorating disease severity in cutaneous leishmaniasis caused by *L*. *braziliensis* infection.

## Author summary

Cutaneous leishmaniasis is a neglected tropical disease, with over 200,000 new cases reported annually. The disease caused by *L*. *braziliensis* leads to chronic ulcerated lesions that often fail conventional treatment, and thus, new therapies to control the disease are needed. One of the major factors promoting increased disease and treatment failure is the recruitment of cytolytic T cells to the cutaneous lesions, which promotes increased inflammation without controlling the parasites. This study explores the role of the innate receptor NKG2D in *L*. *braziliensis* patients. Using clinical samples from *L*. *braziliensis* patients, we demonstrate that NKG2D is expressed in leishmanial lesions, identify factors that promote NKG2D and NKG2D ligand expression, and find that NKG2D gene expression is associated with treatment failure. Based on these results, we propose that targeting NKG2D might be considered as a therapy to dampen disease severity in *L*. *braziliensis* patients.

## Introduction

Cutaneous leishmaniasis has a broad spectrum of clinical manifestations ranging from relatively mild to chronic and severely disfiguring clinical forms. Infection with *L*. *braziliensis* causes particularly severe cutaneous lesions, mainly due to an uncontrolled inflammatory response [1]. Several studies have correlated the presence of CD8 T cells with an increased disease in *L*. *braziliensis* patients [2–5]. Using mouse models and transcriptional studies of patients’ lesions, we demonstrated that cytotoxicity induced by CD8 T cells drives immunopathology in cutaneous leishmaniasis [6–10]. Cell death in leishmanial lesions led to inflammasome activation and IL-1β release, thereby promoting inflammation and disease severity [5,6]. In addition to the specific killing of leishmania-infected target cells, we found that CD8 T cells activated by antigen-independent mechanisms referred to here as bystander CD8 T cells can also play a detrimental role in experimental cutaneous leishmaniasis [8,9]. Whether bystander CD8 T cells play a similar role in human cutaneous leishmaniasis is unknown.

Bystander CD8 T cells can be either beneficial or detrimental in disease outcome, depending on the infection model or disease [9,11]. For example, bystander CD8 T cells can provide a protective response against pathogens [12,13]. Other findings demonstrated that bystander CD8 T cells could have a harmful contribution, promoting disease severity. For example, in hepatitis A infection, hepatitis A-unrelated CD8 T cells mediate cytotoxicity, leading to liver injury [14]. In cutaneous leishmaniasis, we found that a previous murine infection with lymphocytic choriomeningitis virus (LCMV) led to an expansion of CD8 T cells. When the mice were challenged with *Leishmania* 4 weeks later, LCMV-specific CD8 T cells were non-specifically recruited to the leishmanial lesions, promoting increased disease [9]. Moreover, co-infection with LCMV had a similar effect in exacerbating cutaneous lesions [8]. In both cases, CD8 T cell depletion or blockade of NKG2D ameliorated the increased disease, implicating NKG2D expressing CD8 T cells as the mediators of increased pathology [8,9]. These results suggest that your immunological history can significantly influence the outcome of subsequent unrelated infections.

*KLRK1* mRNA encodes NKG2D, an innate receptor expressed on NK cells, γδ T cells, and αβ CD8 T cells [15], and binds to stressed-induced ligands absent or expressed at low levels in normal tissues [16,17]. The ligands for NKG2D in humans belong to the MHC class I chain-related protein A and B (MICA and MICB) and UL16 binding protein (ULBP1-ULBP6) families [18,19] and are induced in sites of inflammation [15,20–24]. In many cases, NKG2D expression in CD8 T cells enhances T cell receptor (TCR) signaling and thereby enhances the killing of specific targets [25]. However, NKG2D ligation in CD8 T cells can also promote CD8 T cell killing without TCR signaling in situations where IL-15 expression in the tissue is high [26,27]. For example, dysregulated IL-15 expression in celiac disease resulted in NKG2D-dependent CD8 T cell killing of target cells and promoted immunopathological responses [27]. Thus, NKG2D might facilitate specific and bystander cytotoxicity in leishmanial lesions.

We show that NKG2D and its ligands are expressed within cutaneous lesions from *L*. *braziliensis*-infected patients. We found that IL-15 promoted increased NKG2D expression to a much greater extent in PBMCs from patients compared to PBMCs from healthy subjects. Similarly, IL-1β enhanced the expression of MICA/B in monocytes from patients but not healthy subjects. Implicating a vital role for NKG2D in *L*. *braziliensis* patients, we show that the blockade of NKG2D in a subset of patients reduces the degranulation of CD8 T cells isolated from leishmanial lesions. Further, we found that *KLRK1* mRNA expression in lesions is associated with treatment failure. These results suggest that NKG2D influences the outcome of infection with *L*. *braziliensis* and that NKG2D is a possible target for immunotherapy.

## Methods

### Ethics statement

This study was conducted according to the principles specified in the Declaration of Helsinki and under local ethical guidelines. This study was approved by the Ethical Committee of the Federal University of Bahia (Salvador, Bahia, Brazil)(010/10) and the University of Pennsylvania IRB (Philadelphia, Pa)(812026;823847). All patients provided written informed consent for collecting samples and subsequent analysis.

### Transcriptional profiling of lesion biopsies from cutaneous leishmaniasis patients

All RNA seq data and clinical metadata from this study are derived from published transcriptional profiling [10]. cDNA library preparation and RNA sequencing were generated from 21 lesion biopsy samples and 7 normal skins. The analyses were performed using the statistical computing environment R version 3.5.1 in RStudio version 1.1.456 and Bioconductor version 3.8 [28]. Transcript quantification data were summarized to genes using the tximport package [29] and normalized using the trimmed mean of M values (TMM) method in edgeR [30]. Data are deposited on the Gene Expression Omnibus (GEO) database for public access (GSE number GSE127831). Microenvironment cell population (MCP)-counter [31] was used to estimate the abundance of cell populations from the RNA-seq data through the immunedeconv package [32]. The analysis carried out for this current study was derived from the normalized gene expression generated previously in [10] and available for download on Gene Expression Omnibus accession number GSE127831. Pearson correlation coefficient was used to determine the correlation between LOG2 expressions of genes from human skin transcripts. Statistical analysis was calculated using GraphPrism v7. Statistical significance was determined using the two-tailed unpaired Student’s t-test, and P < 0.05 was considered statistically significant.

### Lesion biopsies

Before therapy, biopsies were collected at the border of the lesions using a 4 mm punch. Biopsies were treated with 250 mg/mL of Liberase (Roche) for 90 mins at 37°C under 5% CO_2_. Tissue was dissociated using a cell strainer (40 mm, BD Pharmingen), and single-cell suspensions were used for flow cytometric analysis.

### Peripheral blood mononuclear cell cultures

Peripheral blood mononuclear cells (PBMCs) were obtained from heparinized venous blood layered over a Ficoll-Hypaque gradient (GE Healthcare), then washed by centrifugation and resuspended in RPMI 1640 media (Gibco) supplemented with 10% fetal bovine serum (FBS) (Gibco), 100 IU/mL penicillin and 100 μg/mL streptomycin (all Invitrogen). PBMCs were adjusted to a concentration of 1 × 10^6^ cells/mL. Cells incubated with 10 ng/mL of recombinant IL-15 (Petrotech), 50 ng/mL of recombinant IL-1β (Peprotech), and 500 ng/mL of lipopolysaccharides (LPS) (Sigma) were incubated for 18h at 37°C under 5% CO_2_.

### Flow cytometric analysis and antibodies

Cell suspensions from human skin or PBMCs were stained with flow cytometry antibodies directly ex vivo. For the degranulation assay, cells from the skin were incubated for 6 hours with anti-CD107a antibody (BD Pharmingen) and Monensin with or without 25 μg/mL anti-NKG2D blocking antibody (R&D Systems) followed by surface staining. Antibodies: anti-CD11b APC-eFluor 780 (clone ICRF44), anti-CD8a PeCy5.5 (clone 53–6.7), anti-CD45 Pecy7 (clone HI30), anti-CD8 Percp-cy5.5 (clone 53–6.7), anti-TCRα/β APC (clone IP26), anti-NKG2D PE (clone 1D11), anti-MICA/B eF488 (clone 6D4) (all eBioscience), and anti-CD14 BV711(clone 63D3) from Biolegend. All flow cytometry analysis was performed using the FlowJo Software.

### Immunohistochemistry

Samples were fixed in formaldehyde and embedded in paraffin. Deparaffinization and rehydration of 5-μm thick sections were performed using xylene and alcohol. Antigen retrieval was performed using citrate buffer Ph 6.0 at 96°C, and samples were blocked with hydrogen peroxide and Protein Block Serum-Free (DAKO). The slides were incubated overnight at 4°C with the antibody Monoclonal Mouse Clone 159207 (R&D). Mouse and Rabbit Peroxidase Kit/HRP KP500 (Diagnostic BioSystems) were used according to the manufacturer’s instructions. All slides were counterstained with Harris hematoxylin, dehydrated, and mounted. Images were captured using an optical microscope attached to a digital camera system, and images were analyzed by Image-Pro Plus (Media Cybernetics). The number of positive cells was quantified in five randomized fields using the selection feature and semiautomatic counter of 1.48v ImageJ software (National Institutes of Health).

### Statistical analysis

Statistical significance was determined using the two-tailed unpaired Student’s *t*-test, except for the paired t-test used for human experiments in which the same patient sample was compared between different treatments or between PBMC and the skin of the same patient. Differences were considered significant when *p* ≤ 0.05 (*), *p* ≤ 0.01 (**), *p* ≤ 0.001 (***), or *p* ≤ 0.0001 (****).

## Results

### KLRK1 *is expressed in lesions and is correlated with cytolytic gene expression*

**Fig 1:**
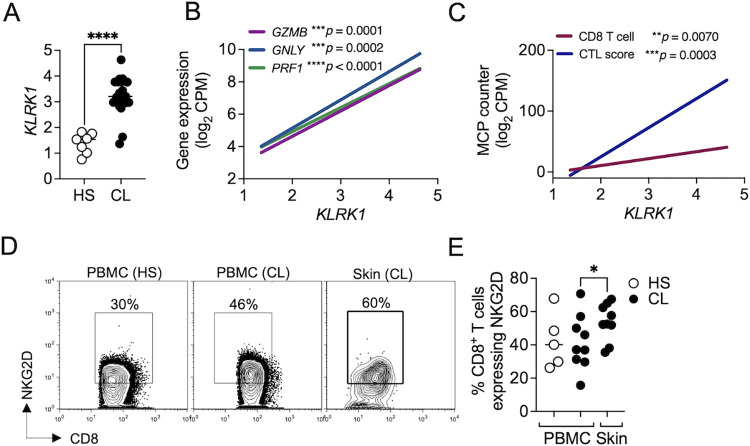
*KLRK1* is expressed in lesions and is correlated with cytolytic genes. RNAseq analysis from 7 healthy skin (HS) and 21 lesions from *L*. *braziliensis*-infected patients (CL). (A) Gene expression of *KLRK1*, which encodes NKG2D. (B) Correlation shows *KLRK1* expression compared to *GZMB*, *GNLY*, and *PRF1*. (C) Correlation shows *KLRK1* expression compared to MCP counter abundance scores for CD8 T cell and CTL score. Gene expression is represented as counts per million (CPM) in the log2 scale. (D and E) Cells isolated from lesions or PBMC obtained from HS and *L*. *braziliensis* patients were stained for flow cytometry directly *ex vivo* and depicted are representative flow cytometry plots (D) and scatter plots (E) of NKG2D expression in CD8 T cells. Data were obtained from 5 HS and 9 *L*. *braziliensis* patients. HS, healthy subjects; CL, cutaneous leishmaniasis; PBMC, peripheral blood mononuclear cells. *p<0.*05*. *p < 0.*05*, **p ≤ 0.*01*, ***p ≤ 0.*001*, ****p < .*0001*.

We previously demonstrated that NKG2D engagement on bystander CD8 T cells promotes pathology in experimental models of cutaneous leishmaniasis [8,9]. To determine whether NKG2D plays a role in cytotoxicity induced by CD8 T cells in *L*. *braziliensis-*lesions, we first determined if NKG2D is expressed in *L*. *braziliensis* lesions. Using an RNAseq dataset of 21 skin biopsies from cutaneous leishmaniasis lesions and 7 from healthy individuals [10], we found that *KLRK1*, which encodes NKG2D, is enriched in lesions of patients compared to healthy skin (Fig 1A). We next investigated if *KLRK1* expression was associated with cytolytic gene expression and found a strong correlation between *KLRK1* expression and *GZMB*, *GNLY*, and *PRF1* in the lesion (Fig 1B). We then correlated the expression levels of *KLRK1* and the abundance of cytotoxic CD8 T cells in lesions using MCP-counter, a computational pipeline based on gene markers that predicts cell type abundances from bulk RNA-seq samples [31]. We found a strong correlation between *KLRK1* expression and the quantity of both CD8 T cells and cytolytic lymphocytes at lesions (Fig 1C).

To address whether CD8 T cells in *L*. *braziliensis* lesions express NKG2D, we collected skin biopsies from *L*. *braziliensis*-infected patients and PBMC from the same individuals and obtained PBMC from healthy subjects. By analyzing the expression of NKG2D by flow cytometry, we found that circulating CD8 T cells express NKG2D similarly between healthy subjects and *L*. *braziliensis-*infected patients. There was a small but significant increase in NKG2D-expressing CD8 T cells in *L*. *braziliensis* lesions compared to CD8 T cells in the blood (Fig 1D and 1E). However, the important observation is that CD8 T cells in the lesion express NKG2D. Together, these data demonstrated that NKG2D is expressed by CD8 T cells in *L*. *braziliensis* lesions and is correlated with cytolytic machinery components.

### *NKG2D ligands are expressed in lesions from* L. braziliensis*-infected patients*

**Fig 2:**
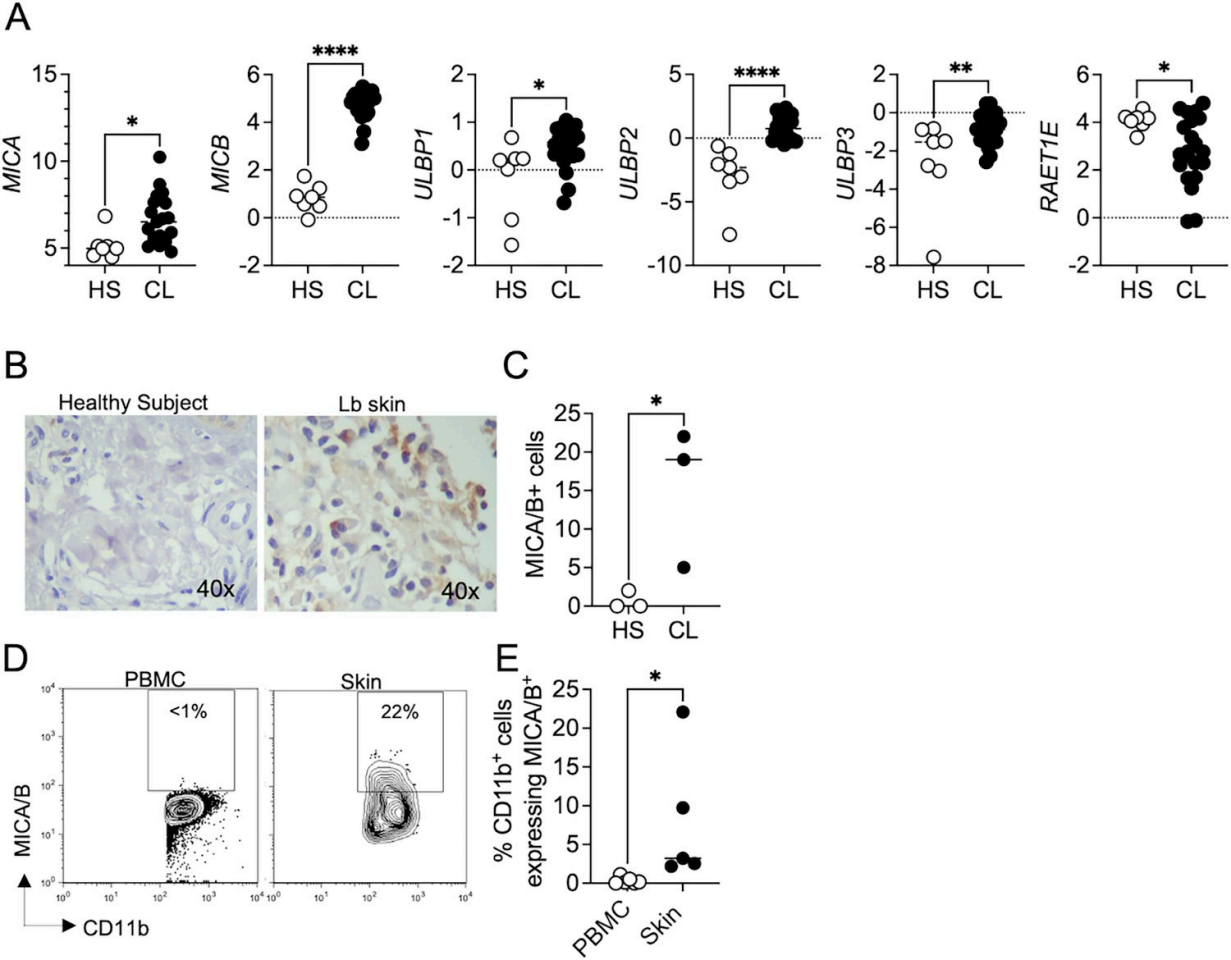
NKG2D ligands are enriched in lesions from *L*. *braziliensis*-infected patients. RNAseq analysis from 7 healthy skin (HS) and lesions from 21 *L*. *braziliensis*-infected patients (CL). (A) Gene expression of *MICA*, *MICB*, *ULBP1*, *ULBP2*, *ULBP3*, and *RAET1E* (ULBP4). (B and C) Immunohistochemistry for MICA/B in biopsies from *L*. *braziliensis* patients’ lesions. Data were obtained from 3 HS and 3 *L*. *braziliensis* lesions. (D and E) Cells isolated from lesions or PBMC obtained from *L*. *braziliensis* patients were stained for flow cytometry directly *ex vivo* and depicted are representative flow cytometry plots (D) and scatter plots (E) of MICA/B expression in CD11b^+^ cells. Data were obtained from 6 PBMC and 5 skin lesions. HS, healthy skin; CL, cutaneous leishmaniasis; PBMC, peripheral blood mononuclear cells. *p < 0.*05*, **p ≤ 0.*01*, ***p ≤ 0.*001*, ****p < .*0001*.

By analyzing our RNAseq data set, we found that the NKG2D ligands, *MICA*, *MICB*, *ULBP1*, *ULBP2*, *ULBP3*, and *RAET1E*, were enriched in lesions of patients compared to healthy skin (Fig 2A). We also observed increased MICA/B protein expression in lesions from patients by immunohistochemistry and flow cytometry (Fig 2B–2E). The expression of MICA/B was specific to the lesions of *Leishmania*-infected patients, as normal skin did not express MICA/B (Fig 2C and 2D). We found that MICA/B expression in CD11b^+^ cells was only detected in the skin of *L*. *braziliensis* patients and not in the blood (Fig 2D and 2E), suggesting that NKG2D-ligand expression is specific to the skin and not a systemic response to infection.

### *IL-15 induces NKG2D on CD8 T cells from* L. braziliensis*-infected patients*

**Fig 3:**
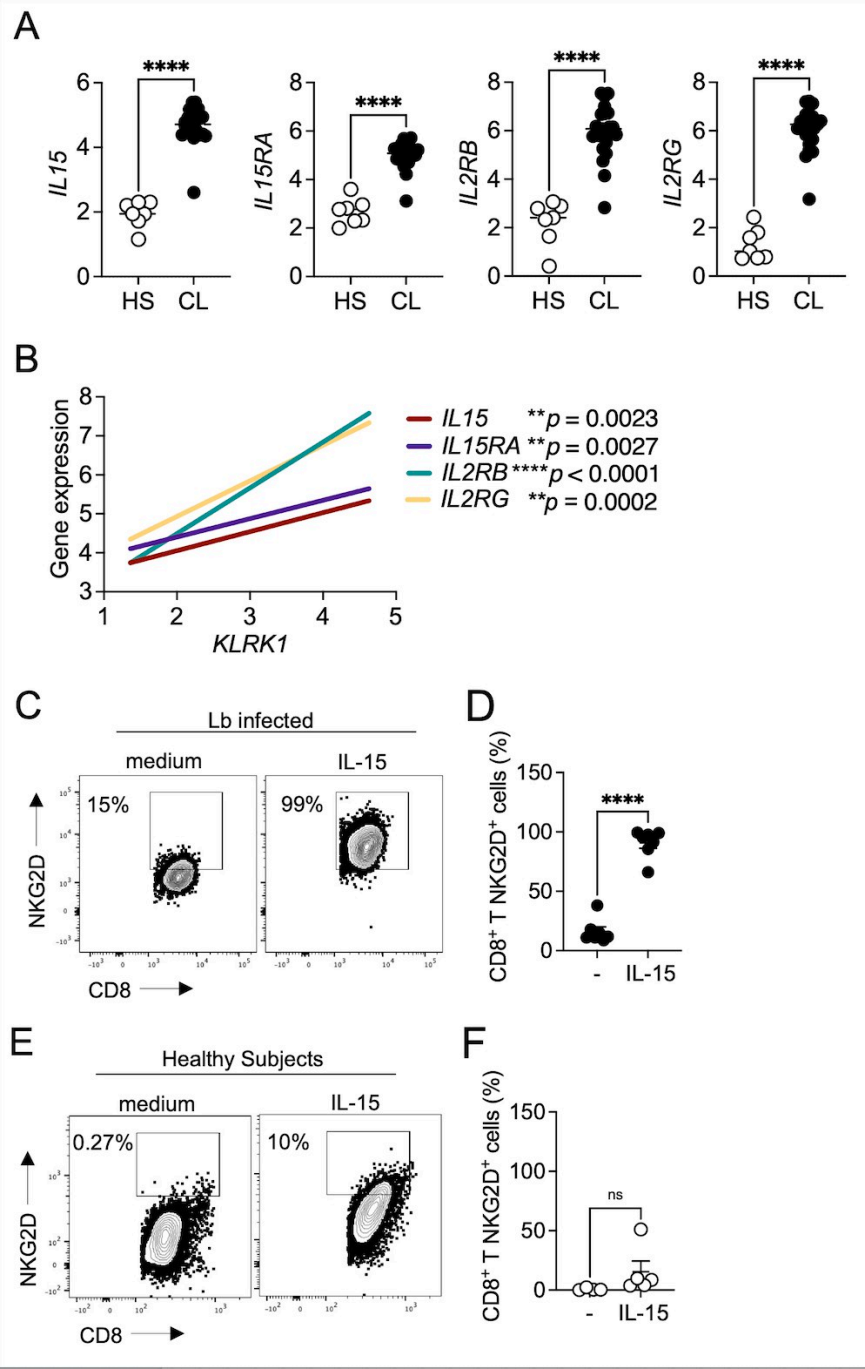
IL-15 induces NKG2D expression on CD8 T cells from *L*. *braziliensis*-infected patients. RNAseq analysis from 7 healthy skin (HS) and lesions from 21 *L*. *braziliensis*-infected patients (CL). (A) Gene expression of *IL15*, *IL15RA*, *IL2RG*, and *IL2RG* in the skin of HS and lesions from *L*. *braziliensis*-infected patients. (B) Correlation between *KLRK1* expression and *IL15*, *IL15RA*, *IL2RB*, and *IL2RG* expression in the lesions. Gene expression is represented as counts per million (CPM) in the log2 scale. (C–F) PBMCs from *L*. *braziliensis*-infected patients and healthy subjects were cultured with IL-15 for 18h and stained for flow cytometry. Dot plots (C and E) and graph bars represent (D and F) NKG2D expression by CD8 T cells after IL-15 stimulation. Data were obtained from 5 healthy subjects and 7 *L*. *braziliensis-*infected patients. HS, healthy skin; PBMC, peripheral blood mononuclear cells. **p ≤ 0.*01*, ***p ≤ 0.*001*, ****p < .*0001*.

IL-15 contributes to CD8 T cell cytotoxicity and has been implicated in upregulating NKG2D expression [26,27,33,34]. Our group previously demonstrated that pharmacological inhibition of IL-15 signaling by tofacitinib treatment ameliorates the pathology of leishmania lesions in an experimental model by suppressing CD8 T-cell cytotoxic potential [35]. To investigate whether IL-15 enhances NKG2D expression in CD8 T cells, we first evaluated the expression levels of IL-15 and its receptors IL-15Rα, which is essential for IL-15 trans presentation to IL-2/15RβƔ. The RNAseq analysis demonstrated the enrichment of *IL15*, *IL15RA*, *IL2RB*, and *IL2RG* in the *L*. *braziliensis* lesions compared to healthy skin (Fig 3A). Additionally, the expression of *KLRK1* was correlated with *IL15*, *IL15RA*, *IL2RB*, and *IL2RG* in the lesions (Fig 3B).

Next, to assess whether IL-15 enhances NKG2D expression on CD8 T cells in cutaneous leishmaniasis, PBMCs from *L*. *braziliensis*-infected patients or healthy subjects were cultured in the presence or absence of IL-15 for 18 hours. We observed that IL-15 stimulation enhanced the frequency of CD8 T cells expressing NKG2D from *L*. *braziliensis*-infected patients (Fig 3C and 3D). No differences were observed in healthy subjects (Fig 3E and 3F). Together, these results demonstrate that IL-15 enhances NKG2D expression by CD8 T cells in *L*. *braziliensis*-infected patients.

### IL-1β induces MICA/B expression in cells from cutaneous leishmaniasis patients

**Fig 4:**
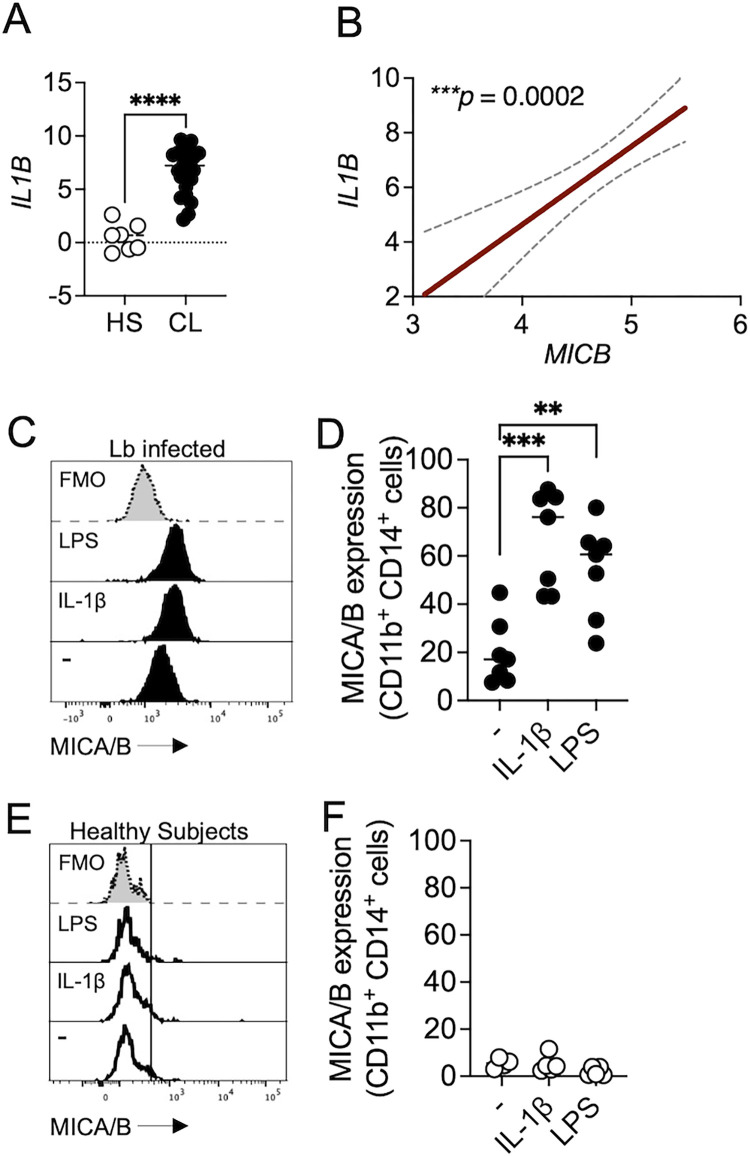
IL-1β induces MICA/B expression in *L*. *braziliensis* patients. RNAseq analysis from 7 healthy skin (HS) compared to lesions from 21 *L*. *braziliensis*-infected patients. (A) Gene expression of *IL1B* in HS and lesions from *L*. *braziliensis*-infected patients (CL). (B) Correlation between *MICB* expression and *IL1B* expression at the lesion. Gene expression is represented as counts per million (CPM) in the log2 scale. (C–F) PBMCs from *L*. *braziliensis* infected patients (Lb) (C and D) or healthy subjects (E and F) were cultured in the presence of IL-1β recombinant for 18h. LPS was used as a positive control of MICA/B induction. Data were obtained from 7 *L*. *braziliensis* infected patients and 5 healthy subjects. Representative histogram of MICA/B expression of *L*. *braziliensis* infected patients (C) and healthy subjects (E) gated in CD11b^+^ CD14^+^ cells. PBMC, peripheral blood mononuclear cells. **p ≤ 0.*01*, ***p ≤ 0.*001*, ****p < .*0001*.

NKG2D ligands are induced in inflammatory tissues in part due to high levels of cytokines [21,22,36], one of which is IL-1β [24]. *L*. *braziliensis*-lesions have a high expression of *IL1B* compared to healthy skin (Fig 4A), and *MICB* expression positively correlated with *IL1B* expression in lesions (Fig 4B). To test if IL-1β enhances the expression of MICA/B, PBMCs from *L*. *braziliensis*-infected patients or healthy subjects were cultured in the presence or absence of IL-1β for 18 hours. We found that IL-1β enhanced the frequency of CD11b^+^ CD14^+^ cells expressing MICA/B in *L*. *braziliensis*-infected patients (Fig 4C and 4D), while no differences were observed in healthy subjects (Fig 4E and 4F). Together, these results demonstrate that IL-1β enhances MICA/B expression on CD11b^+^ CD14^+^ cells in *L*. *braziliensis*-infected patients.

### NKG2D promotes CD8 T cell degranulation, and its expression correlates with treatment failure

**Fig 5:**
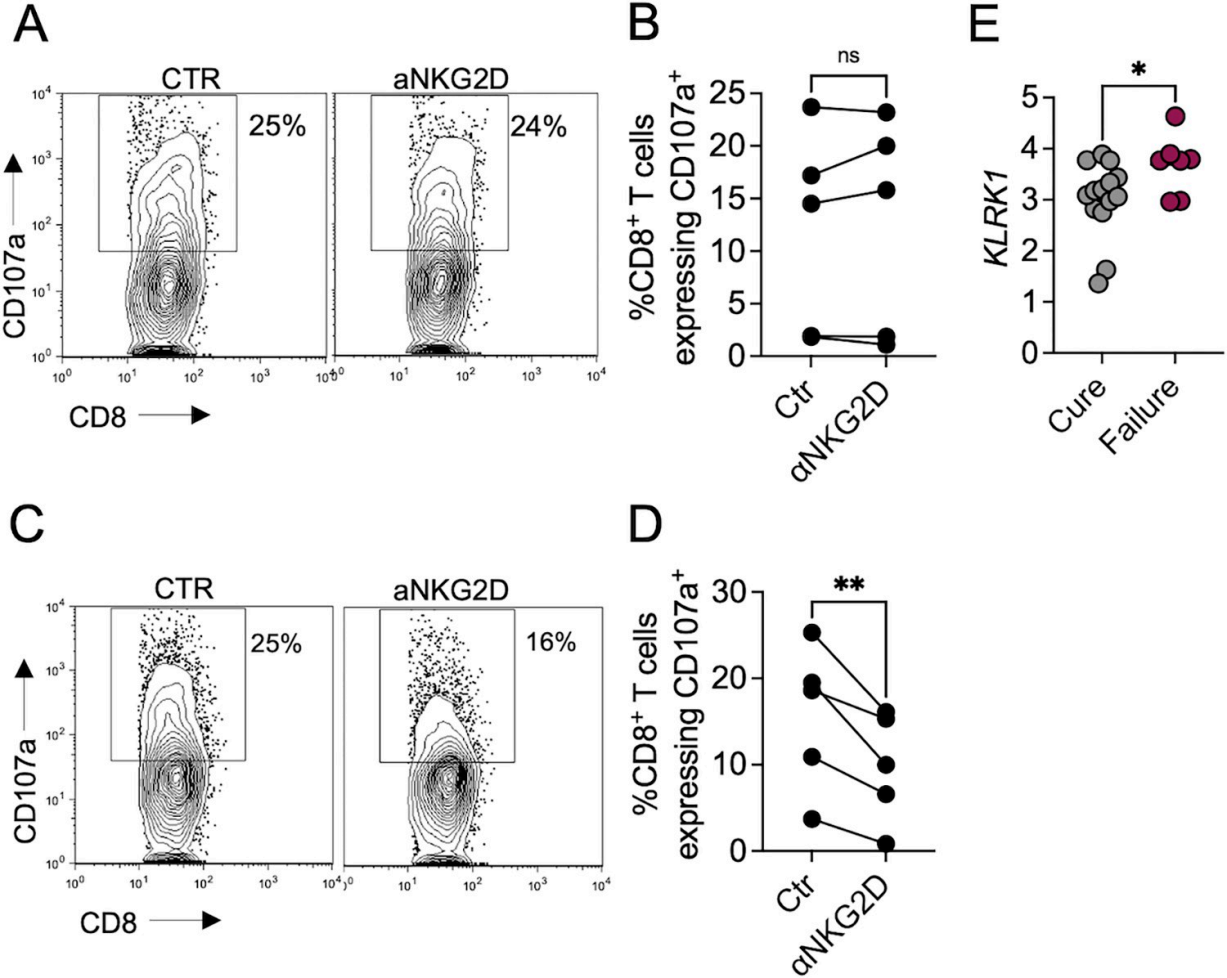
NKG2D promotes CD8 T cell degranulation in a subset of *L*. *braziliensis*-infected patients and is associated with treatment failure. Cells isolated from the lesions of *L*. *braziliensis* patients were incubated with anti-CD107a and cultured with or without anti-NKG2D antibody. Flow cytometry plots (A and C) and scatter plots (B and D) of CD107a expression on the surface of CD8 T cells are depicted. Data were obtained from 10 *L*. *braziliensis*-infected lesions. (E) *KLRK1* log2 counts per million (log2 CPM) expression in patients that cured (n = 14) or failed (n = 7) the first round of treatment with antimony. *p < 0.*05*, **p<0.*01*.

To determine if NKG2D contributed to CD8 T cell cytotoxicity, we tested the role of NKG2D in an assay where CD107a expressed on the surface of the CD8 T cell is assessed as an indication of degranulation [37,38]. Cells from the biopsies were cultured *in vitro* for 6 hours in the presence of anti-CD107a antibody with or without anti-NKG2D blocking antibody, followed by cell surface staining for flow cytometric analysis. We found that degranulation was independent of NKG2D in half of the patients since anti-NKG2D treatment did not affect the degranulation of CD8 T cells (Fig 5A and 5B). However, in 50% of the patients, NKG2D blockade reduced degranulation by 20–80% (Fig 5C and 5D). Since the degranulation assay only reflects the events happening within the 6 hours of the assay, the total NKG2D-dependent degranulation we observed may be an underestimate of *in vivo* events.

Finally, to evaluate if *KLRK1* expression in lesions is associated with treatment failure, we again used our RNAseq data set from patients’ biopsies [10]. Biopsies from lesions were obtained on the day of diagnosis, and the patients were subsequently treated with pentavalent antimony for 21 days. At day 90, patients with complete re-epithelization were considered cured, and patients with active lesions were classified as treatment failures [10]. We found that *KLRK1* expression is higher in patients who failed treatment than those who were cured (Fig 5E). Taken together, we conclude that NKG2D participates in CD8 T cell cytotoxicity in human cutaneous leishmaniasis.

## Discussion

Bystander CD8 T cells are present in human *Leishmania*-infected lesions, although whether they play a role in promoting pathology in an NKG2D-dependent manner, as observed in experimental leishmaniasis [8,9], is unknown. Here, we show that NKG2D and its ligands are expressed in lesions from *L*. *braziliensis*-infected patients. We describe the potential role of IL-15 and IL-1β in promoting lysis mediated by NKG2D-NKG2D ligand interactions. Notably, we found that blockade of NKG2D expression reduced degranulation by CD8 T cells in a subset of patients, and our transcriptional study demonstrated that the higher levels of *KLRK1* expression in the lesion are associated with treatment failure. These results indicate that NKG2D contributes to the pathology and treatment failure in human cutaneous leishmaniasis caused by *L*. *braziliensis* infection.

NKG2D-NKG2D ligand interactions mediate increased cytotoxicity that can be protective against tumors or infections but can also lead to enhanced immunopathology. Tumors and some viruses block this pathway, demonstrating its important role in protection [39–42]. However, it can also be detrimental, as is evident in some autoimmune diseases [43,44]. For example, blocking NKG2D limits autoimmune diabetes in NOD mice [45]. NKG2D mediates these effects by enhancing TCR signaling [25], and thus, it is possible that in leishmaniasis, increased NKG2D-NKG2D ligand interactions promote increased cytolysis within lesions by leishmania-specific T cells. Alternatively, we found in previous studies that NKG2D was required for the ability of bystander CD8 T cells to lyse targets and promote pathology in an experimental murine model [8,9], raising the possibility that NKG2D might also promote killing in lesions from patients in a non-specific manner. Inflammatory sites recruit both specific and non-specific T cells from the circulation, and in human leishmaniasis, *Toxoplasma*-specific T cells were previously described in lesions [46]. Thus, NKG2D-dependent cytotoxicity in humans could be due to specific T cell cytolysis, non-specific bystander CD8 T cell cytolysis, or both.

One situation where NKG2D acts in the absence of cognate antigen recognition is in the presence of high amounts of IL-15 [27], and our transcriptional study revealed enrichment in IL-15-signaling in lesions from *L*. *braziliensis* patients. We found a correlation between IL-15 and *KLRK1* expression at the lesion site and that IL-15 enhances NKG2D expression on CD8 T cells from patients. In agreement with our findings, IL-15 enhances the cytotoxicity of NKG2D^+^ CD8 T cells in other diseases, such as alopecia [34], celiac disease [27], and acute hepatitis A infection [14].

NKG2D ligands are found on several myeloid cells, and their expression is highly regulated. For example, the treatment of human melanoma cells with IFN-γ reduces MICA levels [21], and TGF-β downregulates the transcription of MICA, ULBP2, and ULBP4 in human malignant gliomas [22]. In contrast, IFN-α enhances MICA expression on dendritic cells [23]. Our transcriptional study of *L*. *braziliensis* lesions reveals a strong correlation between *IL1B* and MICB expression. We found that IL-1β enhanced MICA/B expression in the myeloid cell of *L*. *braziliensis*-infected patients. Interestingly, no induction was observed on cells obtained from healthy subjects. Consistent with our results, IL-1β enhances the production of soluble MICA in human hepatocellular carcinoma, but no effect was observed in treated normal hepatocytes [24]. Since *IL1B* is among the most highly expressed genes in leishmanial lesions [10], we hypothesize that IL-1β helps to promote NKG2D ligands in leishmanial lesions.

Our results suggest that pharmacological targeting of NKG2D or IL-15 might benefit patients. In autoimmune and inflammatory diseases where NKG2D plays a pathological role, treatment with a monoclonal antibody has been described as a possible therapy. For example, alopecia areata is driven by NKG2D^+^ CD8 T and is reversed by Jak1/3 inhibition [34,47]. Similarly, the blockade of NKG2D during the pre-diabetic stage in NOD mice prevented the development of diabetes because it abrogated the function of autoreactive CD8 T cells [45]. We previously found that blocking NKG2D or blocking Jak1/3 signaling in a mouse model of leishmaniasis ameliorated severe disease [8,9,35]. We now show that NKG2D expression is associated with treatment failure in *L*. *braziliensis* patients. Thus, these results suggest that therapeutics inhibiting NKG2D signaling might be helpful in dampening disease severity induced by CD8 T cells in cutaneous leishmaniasis caused by *L*. *braziliensis* infection.
